# Transformer-based prediction of radiotherapy couch shift risk in prostate cancer based on rectal volume

**DOI:** 10.1038/s41598-026-42276-7

**Published:** 2026-03-15

**Authors:** Andras Kedves, Tamás Ungvári, Aba Lőrincz, Judit Magyar-Lengyel, József Horváth, Zsófia Dankovics, Döme Szabó, Balázs Kiss, Emerencia Kedves, Dávid Sipos, Judit Olajos, Károly Tőkési

**Affiliations:** 1 Markusovszky Teaching Hospital of County Vas, Markusovszky Street 5, Szombathely, 9700 Hungary; 2 Institute of Center for Medical Physics and Biomedical Engineering, Medical University of Vienna, University of Vienna, Spitalgasse 23, Wien, 1090 Austria; 3 Department of Thermophysiology, Institute for Translational Medicine, Medical School, University of Pécs, Szigeti Street 12, Pécs, 7624 Hungary; 4 József Cziráki Doctoral School of Wood Sciences and Technologies, University of Sopron, Bajcsy-Zsilinszky Street 4, Sopron, 9400 Hungary; 5 Department of Medical Imaging, Faculty of Health Science, University of Pécs, Guba Sándor Street 40, Kaposvár, 7400 Hungary; 6 University of Nyíregyháza, Sóstói Street 31/B, Nyíregyháza, 4400 Hungary; 7 Jósa András University Teaching Hospital, Szent Istvan Street 68, Nyíregyháza, 4400 Hungary; 8 HUN-REN Institute for Nuclear Research, Bem Square 18/c, Debrecen, 4026 Hungary

**Keywords:** Deep learning, Artificial intelligence, Transformer, Prostate, Cancer, Cancer, Computational biology and bioinformatics, Medical research, Oncology

## Abstract

Accurate tumor targeting is vital in prostate cancer (PCa) radiotherapy for precise dose delivery and minimizing healthy tissue exposure. Currently, no artificial intelligence (AI) tool exists to predict setup degradation risk considering rectal volume (RV) changes pre-treatment. Our study aimed to assess this risk and identify a critical RV cutoff. We retrospectively analyzed 498 cone-beam computed tomography (CBCT) scans from 38 PCa patients. Rectal organs at risk were contoured, and 3D couch shifts were calculated. A novel unsupervised 2-stage transformer-based encoder processed longitudinal RV and displacement data. K-means clustering grouped patients, visualized with T-distributed Stochastic Neighbor Embedding (t-SNE). Statistical analysis, including ROC AUC, identified an optimal planning CT RV cutoff. Three distinct patient clusters were observed in CBCT scans. A significant correlation (*p* < 0.001) was found between initial RVs and these clusters. The ROC AUC was 0.93, establishing an optimal cutoff of 81.62 cm³ for planning CT RV, effectively distinguishing patients prone to significant positional variability. Our study developed an AI-driven model predicting patient couch shifts in PCa radiotherapy, identifying a crucial RV threshold. These findings may advance positioning accuracy, enabling proactive adjustments and optimizing therapy.

**Supplementary Information:**

The online version contains supplementary material available at 10.1038/s41598-026-42276-7.

## Introduction

Approximately 1.5 million new cases of prostate cancer (PCa) are diagnosed annually across the globe. The majority of patients, approximately 75%, present with disease localized to the prostate, a stage associated with an excellent 5-year survival rate of nearly 100%. Management strategies for localized disease vary based on progression risk and may include active surveillance, prostatectomy, or radiation therapy^1^.

Prostate adenocarcinoma is curable when detected in its early stages, and its associated symptoms or patient complaints can be effectively managed through appropriate therapeutic interventions. PCa incidence rises with advancing age, and its development is also influenced by both genetic predispositions and ethnic backgrounds. Treatment modalities may encompass surgical removal, hormonal manipulation, chemotherapy, or radiotherapy, with the choice depending on the disease’s stage and other pertinent factors^2–4^.

Focusing on radiotherapy, accurate patient positioning is essential. It ensures that the radiation precisely targets the tumor, thereby protecting healthy surrounding tissues from unnecessary exposure^5^. Clinicians must account for variations in prostate position between treatment fractions, primarily due to changes in bladder and rectal filling^6^. Prescribed diet aimed to prevent both gastrointestinal discomfort, such as bloating, and significant rectal filling, which can lead to prostatic displacement^7^. Maintaining a consistent rectal volume (RV) and, consequently, a stable prostate position, is essential for accurate dose delivery, thereby reducing the incidence of acute and late side effects^8–11^. Beyond dietary management, meticulous patient immobilization is fundamental. Accurate patient placement facilitates a reduction in the size of safety margins around the target volume, consequently decreasing the radiation dose to surrounding organs at risk. This precision helps to prevent hospitalizations stemming from potential side effects^12,13^. Effective interfraction motion treatment, coupled with good nutrition, also could play a significant role^14,15^.

Artificial intelligence (AI) is becoming more and more accepted in clinical data management. In oncoradiology setting machine learning (ML)^6^, deep learning (DL), and novel transformer-based models are showing high potential in classification, segmentation, and prediction^16–19^. PCa predictions^20^ and organ at risk segmentation^21^ are few, but not limited applications of these special AI tools.

An AI-based prediction for possible displacement of the patient on a Linear Accelerator (LINAC) couch before radiotherapy planning does not exist according to our current knowledge. Therefore, in our study, we aimed to identify a cutoff or threshold for rectal volume measured during the initial simulation CT—that reliably indicates an elevated risk of degraded setup accuracy (positional error) during subsequent radiation treatment.

## Materials and methods

### Patients, patient preparation

Ethical approval was obtained from the Regional and Institutional Research Ethics Committee of the Markusovszky University Teaching Hospital of Szombathely under protocol No. 14/2022. Reporting of all experimental procedures complied with recommendations in committee based on the International Council for Harmonisation of Technical Requirements for Pharmaceuticals for Human Use (ICH) Guideline for Good Clinical Practice (GCP) Guidelines.

Between June 2020 and February 2025, 38 pathologically confirmed, primary PCa patients were enrolled in the current retrospective study. All patients underwent volumetric modulated arc therapy (VMAT)-, or intensity modulated radiation therapy (IMRT) based planning computed tomography (CT) based up to 78 Gy on the selected Varian (Varian Medical Systems, Palo Alto, CA) LINACs at the Department of Oncoradiology, Markusovsky University Teaching Hospital, Szombathely, Hungary. Patients whose rectum protruded from the cone-beam computed tomography (CBCT) field of view were excluded.

Prior to and during treatment, patients are consistently prescribed a specialized diet, often supplemented with stool softeners or rectal suppositories, to promote regular bowel movements. Patients are positioned supine with fixed, indexed knee and ankle supports to ensure reproducibility and minimize inter-fractional motion. Our institutional protocol mandates that patients consume 500 ml of water 30 min before treatment, ensuring consistent bladder fullness, to further optimize prostate localization and reduce organ motion^22,23^. In addition, accurate management requires the most precise patient positioning possible. Patients were treated in a supine position, and the position of the indexed knee and ankle support was fixed.

### Treatment, cone-beam computed tomography time-series

Planning CT scans were performed with a SOMATOM go. Sim CT (Siemens Healthineers, Erlangen, Germany) scanner. In general, we used a 7–9 field IMRT (Intensity Modulated Radiation Therapy) or a 3-arc VMAT design using filter-free (FFF) or filtered 6 megavolts (MV) photon radiation determined by the charasteristics of the Planning Target Volume (PTV) and then narrowed the fields to the seminal vesicles.

Our protocol was to perform a CBCT every five sessions to eliminate random errors. To avoid systematic errors, a CBCT was performed in the first three sessions, the average of the displacements was calculated in the fourth session and used to adjust the table position, and then a CBCT was performed again. After January 2024 all patients underwent daily CBCT.

### Organ at risk (OAR) definition

**Fig. 1:**
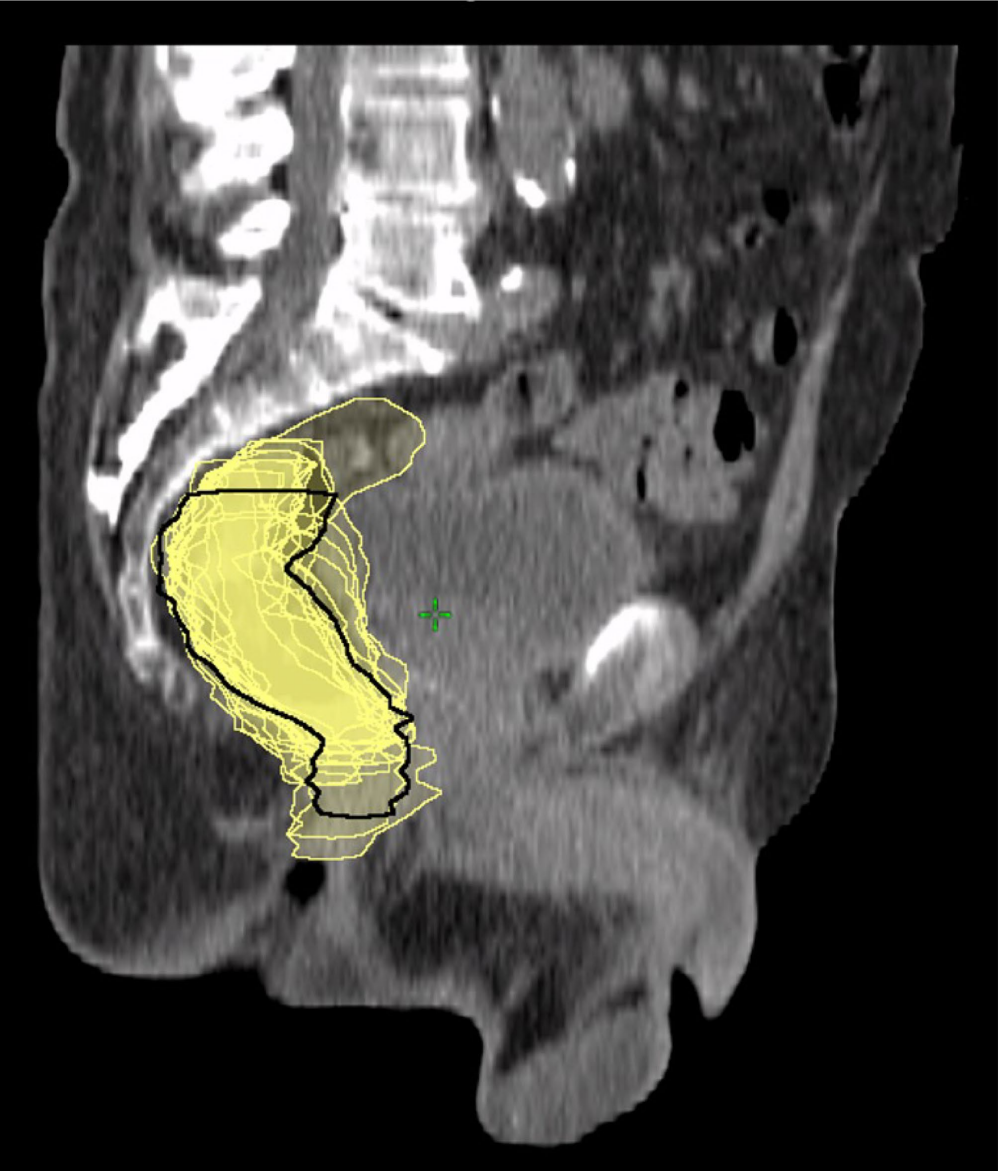
Contoured cone-beam computed tomography (CBCT) structure set overlaid on the planning computed tomography (CT) image. A black contour represents the rectum on the planning CT, while the yellow contours represent the rectum as delineated on the CBCT scans.

Organ at risk (OAR) contouring was performed manually on all CBCT images using the Aria oncology information system (Eclipse v16, Varian Medical Systems, Palo Alto, CA). Rectum contours, extended from the rectosigmoid junction cranially to the proximal anorectal sphincter caudally. A qualified radiation therapy technologist (RTT) with 8 years of experience delineated the rectum using AI-based autosegmentation (v.1.8 Limbus Contour, Radformation, Regina, Saskatchewan, Canada) and then manually fine-tuned the contours. Volumes were supervised and final approved by a chief physician possessing more than 25 years of clinical experience using a rigorous two-step process (Fig. 1.). Based on the defined OARs, rectal volume (cm³) and rectal volume change $\left(\varDelta RV\right)$ calculated as follows:

$$\varDelta RV=Volum{e}_{CBCT}-Volum{e}_{PlanningCT}$$

### Vectors

Vertical (Vrt), longitudinal (Lng), and lateral (Lat) shifts of the couch after the CBCT were collected from Aria software. Based on the Vrt, Lng, and Lat shifts, we introduced a new variable, called the actual displacement vector, which includes full directional and magnitude information per CBCT. Therefore, each CBCT shift (Vrt, Lng, Lat) could be represented as a 3D displacement vector, which described how much and in which direction the couch moved compared to the planning CT. Displacements were calculated as follows:

$$Displacement=\varDelta Vrt,\varDelta Lng,\varDelta Lat$$

where Δ represents the difference between CBCT scan positions compared to Planning CT.

Then, we calculated the vector magnitude as follows:

$$Magnitude=\sqrt{{\left(\varDelta Vrt\right)}^{2}+{\left(\varDelta Lng\right)}^{2}+{\left(\varDelta Lat\right)}^{2}}$$

### Feature extraction & Clustering: 2-stage model

#### Stage 1: Unsupervised transformer-based model

Our analytical pipeline involved a basic self-developed Transformer-based encoder for patient sequence embedding followed by K-means clustering. This Transformer model was designed to process Lng patient data, specifically “Delta_Rectum_Volume,” “Displacement_Magnitude,” and “Rectum_Volume,” which were standardized and padded to a uniform sequence length. It comprised a linear embedding layer to project input features into a higher-dimensional space (${d}_{model}=64$), a positional encoding component to capture temporal information, and two Transformer encoder layers (${n}_{head}=4$, $nu{m}_{encoder\_layers}=2$, $di{m}_{feedforward}=128$). A schematic illustration of the conceptual neural network can be seen on Supplementary Fig. 1. Self-attention mechanisms were utilized in the encoder to weigh the importance of different time points within each patient’s treatment course, ultimately generating a single embedding vector for each patient by averaging the non-padded tokens.

#### Validation

The entire dataset of 498 CBCT sequences was divided into five non-overlapping folds. In each iteration, 80% of the data served as the Training Set (to update model weights), and the remaining 20% served as the Validation Set (to monitor performance and control for overfitting). The best-performing model (based on the average validation runs using reconstruction loss [Mean Squared Error]) was selected to generate the final patient embeddings.

#### Stage 2: K-mean clustering

**Fig. 2:**
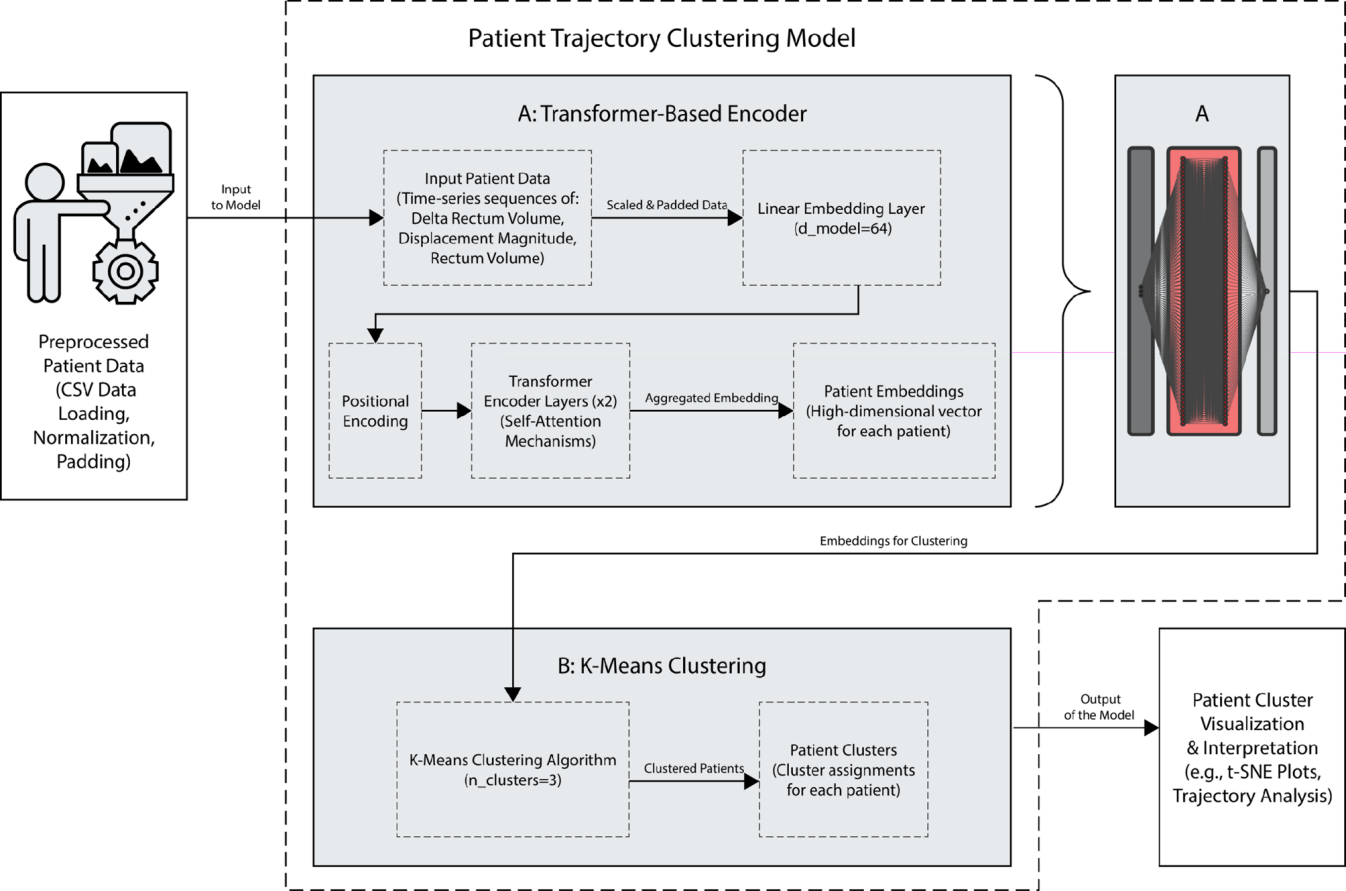
Architecture of the Patient Trajectory Clustering Model. This two-stage deep learning model, which consists of Transformer-Based Encoder (**A**) and K-means Clustering (**B**) stages, and designed to identify distinct patient subgroups based on sequential physiological data. Details of Transformer-Based Encoder can be seen on Supplementary Fig. 1. Details of the Patient Clusters can be seen on Fig. 4.

Subsequently, K-means clustering was applied to these patient embeddings to group patients into distinct cohorts based on the similarity of their daily feature trajectories. Our unsupervised learning approach identified three clusters ($n\_\left\{clusters\right\}=3$), with their distribution visualized using t-distributed Stochastic Neighbor Embedding (t-SNE) in a two-dimensional space. These resulting clusters provided a data-driven segmentation of patients, reflecting diverse response patterns to treatment based on the selected metrics, which we called the Patient Trajectory Clustering Model. Model architecture can be seen in Fig. 2.

### Statistics, visualisations, AI model development

#### Deep learning models

Statistical analysis and AI model development was performed using Python 3.11 (Python Software Foundation, Wilmington, DE, USA). For the Artificial Intelligence model development, the PyTorch library was employed to build a deep neural network model. The architecture included components from torch.nn, such as a Transformer Encoder (TransformerEncoder, TransformerEncoderLayer), for sequence learning and feature extraction. Model training utilized the torch.optim module with the Adam optimizer. Prior to model input, data normalization was performed using the StandardScaler from scikit-learn (sklearn.preprocessing). The resulting high-dimensional feature embeddings were subsequently reduced for visualization using t-distributed Stochastic Neighbor Embedding (t-SNE), provided by the sklearn.manifold library, and clustered into distinct patient groups using the K-Means clustering algorithm from sklearn.cluster.

#### Data analysis, statistics

Data evaluation employed descriptive statistics (mean ± standard deviation) and the interquartile range for variables with non-normal distributions. Shapiro-Wilks tests were performed to assess the normality of the measured RVs and the Vrt, Lng, and Lat offset values. We used the Spearman correlation coefficient to assess the correlation, as the data showed non-normal distributions (*p* < 0.001). Based on the Bonferroni-corrected Mann-Whitney U test, we calculated a cut-off value, which would predict the high table replacement. Thus, we performed a Receiver Operating Characteristic Curve Area Under the Curve (ROC AUC) analysis to determine an optimal cutoff value of the initial RV cm^3^ to identify patients with high baseline RVs. Statistical significance was defined as p values less than 0.05.

#### Visualization

Data processing and manipulation were performed using the pandas and NumPy libraries. The plotting were achieved with Matplotlib (utilizing the mpl_toolkits.mplot3d submodule), seaborn, and was conducted using functions from the SciPy library (plotly.graph_objects). Graphical abstract and advanced visuals were made in Adobe Illustrator CC 2024. (Adobe Inc., San Jose, CA).

## Results

A total of 38 patients with a median age of 74 ± 5.5 years (range: 61–82) and 498 CBCTs were enrolled in this study. The average values for Vrt, Lng, and Lat were − 0.0627, 0.1625, and 0.0444, respectively, with corresponding medians of -0.05, 0.14, and 0.03. Absolute values were − 2.74 for Vrt, -3.81 for Lng, and 3.77 for Lat.

**Fig. 3:**
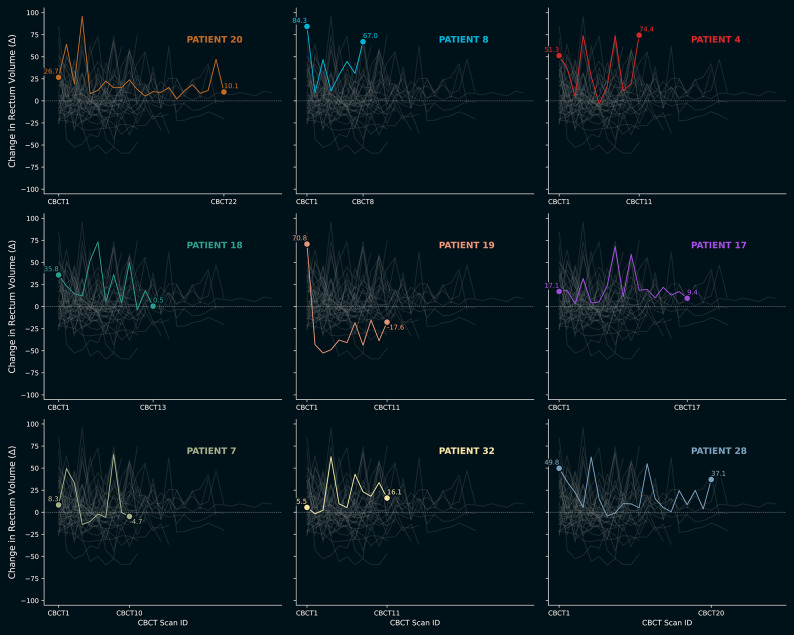
Time-series line plots (in a small-multiples format) depicting the change in rectum volume (Δ) for nine individual patients across multiple Cone-Beam CT (CBCT) scans, relative to the initial Planning CT. The nine patients were specifically selected based on exhibiting the largest absolute displacement magnitude observed during treatment to highlight the range of anatomical variability. The colored lines highlight the displacement values for a specific patient, while the faint lines behind it represent all the patients. The volume change values corresponding to the first and last scan are written out numerically. The horizontal dashed line at y = 0 serves as the reference point corresponding to the planned volume. The figure therefore illustrates the significant volumetric variability observed in a subset of patients throughout the course of radiation therapy. A point close to zero suggests good reproducibility of the planned volume, while points far from zero (either positive or negative) indicate volumetric shifts.

Figure 3 shows time-series line plots depicting the change in ΔRV for nine individual patients across multiple CBCT scans, relative to the initial Planning CT. Average and median RVs were 64.95 and 58.50 cubic centimeters. Both between and within patients line plot has been created to visualize the high RV variability using $\varDelta RV$ throughout treatment.

### Feature extraction & Clustering: Transformer-based model, K-means clustering, t-SNE plot

**Fig. 4:**
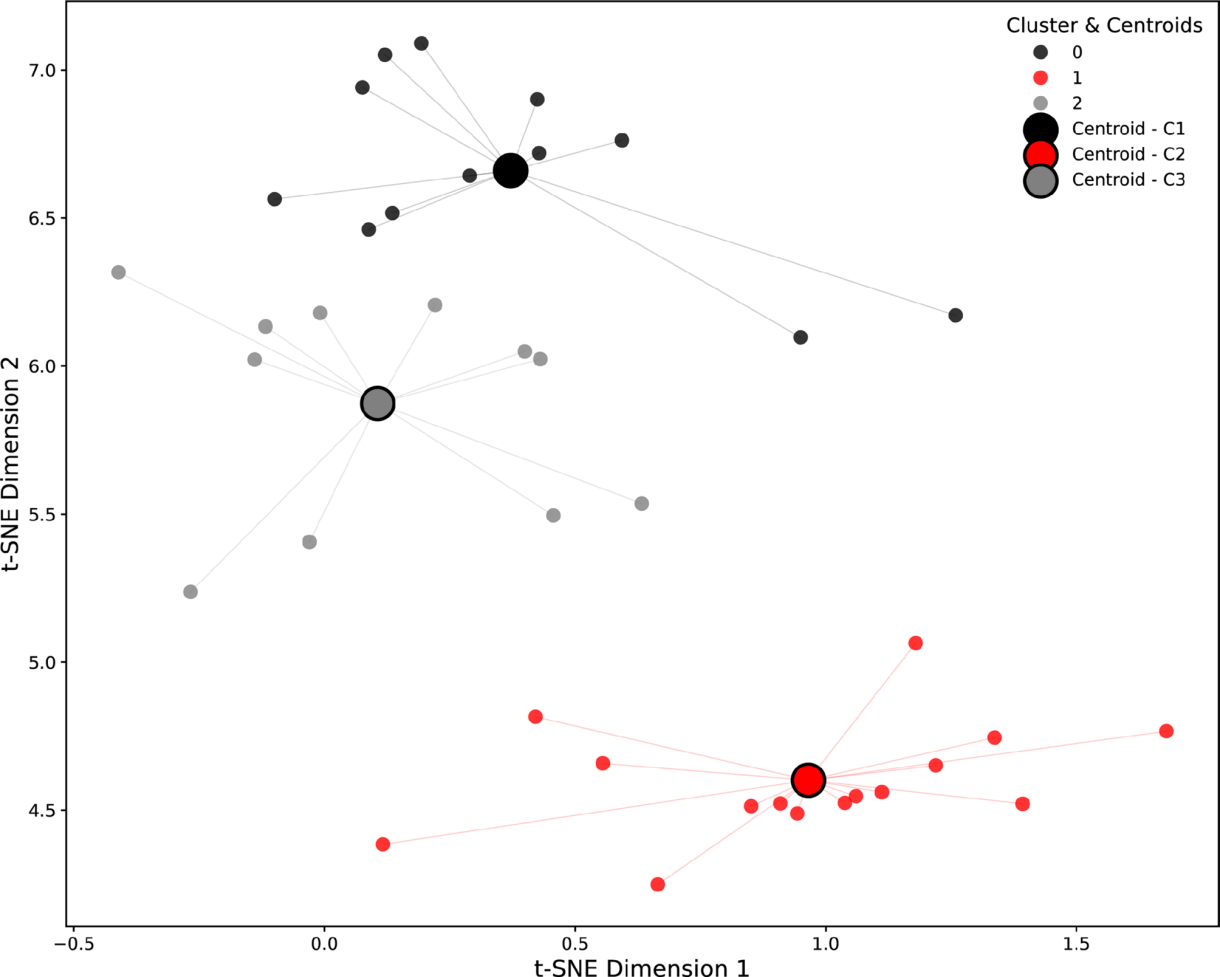
A two-dimensional t-distributed Stochastic Neighbor Embedding (t-SNE) projection of patient-level embeddings, generated by a Transformer encoder from daily CBCT-derived anatomical features. The features analyzed include Delta_Rectum_Volume, Displacement_Magnitude, and Rectum_Volume over the course of radiation therapy. Each point represents a unique patient and centroids, and colors denote the assigned cluster resulting from K-Means clustering (K = 3) applied to the high-dimensional Transformer embeddings. Distinct visual separation between the colored groups indicates the successful identification of heterogeneous patient subgroups characterized by unique sequence patterns of rectal anatomical changes during treatment. These clusters suggest different patient response trajectories relevant to personalized adaptive radiation therapy. C1 – cluster 1, C2 – cluster 2, C3 – cluster 3.

T-SNE plots revealed visually distinct and separated clusters, indicating that the Transformer model successfully identified patient-specific patterns. K-Means clustering subsequently identified meaningful groupings, where patients within the same color-coded cluster exhibited greater similarity in their Lng RV and displacement characteristics compared to those in different clusters. Cluster separation in the t-SNE plot suggests distinct patient subgroups, with well-divided clusters implying clear differences in their underlying patterns. Therefore, no elbow method for refinement was needed. Denser regions within a cluster indicate a higher degree of similarity among patients in that particular area. Furthermore, denser regions within individual clusters signified a high degree of similarity in Lng rectal characteristics among patients belonging to the same cluster, as determined by the K-Means algorithm. (Fig. 4., Supplementary Fig. 2.).

### Prediction

**Fig. 5:**
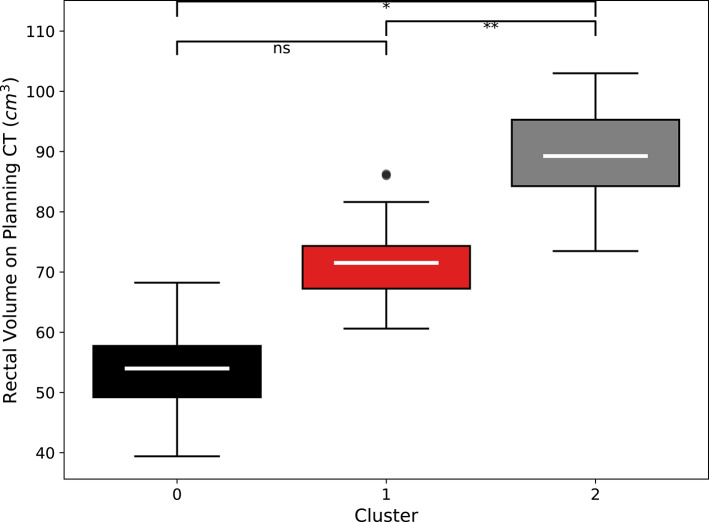
Box plot of initial RV (Planning CT) across the three identified patient clusters, showing significant differences. Asterisks denote statistically significant differences between clusters, determined by the Mann-Whitney U test with Bonferroni correction: * *p* < 0.0001, ** *p* < 0.01, ns non-significant.

After creating clusters, we investigated the connection between RV and the clusters. The significant p-value (*p* = 0.0001) from the Kruskal-Wallis test supports the assertion that differences in initial RVs are linked to the clustering results. ROC-AUC test based on the Bonferroni-corrected Mann-Whitney U tests were applied to investigate how groups are exactly related (Fig. 5.).

### Cut-off value

Calculated ROC AUC = 0.93 (Supplementary Fig. 3.). Using ROC curve analysis with Youden’s index^24^, we determined an optimal cutoff value for Planning CT (the baseline rectum volume measured on planning CT) to distinguish patients with high baseline volumes (Cluster 2) from those with lower values (Clusters 0 and 1). As indicated by Youden’s index, the resulting threshold maximized the sum of sensitivity and specificity, and provided a practical and statistically driven means to classify patients into distinct anatomical subgroups. The cutoff value is 81.62 cm^3^.

## Discussion

Accurate patient positioning is paramount in PCa radiotherapy to ensure precise dose delivery to the target while minimizing exposure to surrounding organs at risk. We developed a system that predicts, from RV measured on the simulation CT, the risk of setup error that could degrade treatment accuracy at irradiation. Furthermore, this system identified a cutoff value for RV at the time of simulation CT. Accurate localization and treatment delivery can help escalate the radiation dose to the target area, leading to improved clinical outcomes^25^.

In our study, patients with an initial rectum volume greater than 81.62 cm^3^ were more likely to fall into Cluster 2, which has been previously shown to have significantly higher baseline rectum volumes compared to Clusters 0 and 1. Consequently, this 81.62 cm^3^ threshold may serve as a decision point for clinicians. For example, patients exceeding this cutoff might be flagged for tailored treatment planning, such as adjustments in table positioning or strategy modifications, based on their higher initial volumes, potentially correlating with differences in treatment response or toxicity profiles.

Integrating our predictive model could represent a shift from reactive quality assurance toward proactive risk mitigation. The model is designed to generate predictions of setup uncertainties, which might allow for immediate, low-cost actions such like minor patient repositioning. This process may help maximize geometric accuracy and could potentially reduce the dosimetric impact of setup errors on healthy tissue.

If initial adjustments are insufficient, the model could provide an essential early warning for a more substantive intervention: re-acquiring the simulation CT scan. The model might signal a deviation from the original planning CT, which could then justify re-scanning to capture the current patient anatomy. Ultimately, the accurate re-scan should facilitate a necessary re-planning process, potentially contributing to the long-term geometric validity of the treatment.

The final clinical decision informed by the predictive model could involve the adjustment of planning margins. For physiological uncertainties that preparation may not resolve, the model might guide the team to either increase margins to improve target coverage or decrease margins to better spare OARs. This data-driven approach could potentially replace empirical recipes with more personalized, risk-adapted strategies, which should help resolve clinical ambiguity.

Our observations indicate an absence of systematic trend-based variations across the cohort (e.g., patterns resembling damped oscillatory motion on Fig. 3.). However, distinct individual variations were noted among specific cases. Analysis of nine selected subjects revealed that Patient 19 exhibited a profile suggestive of stabilization; specifically, rectal filling was significantly elevated during the initial two CBCT scans but decreased substantially in subsequent sessions relative to the baseline CT. This observation may suggest improved dietary compliance over time, though the lack of uniformity across other subjects highlights significant inter-patient variability in physiological consistency.

While our study employed more conventional treatment schemes, the importance of precise patient positioning is further amplified in the context of prostate stereotactic body radiation therapy (SBRT). Ehrbar et al. previously highlighted the dosimetric advantages of motion compensation techniques (e.g. ensuring optimal target coverage and safeguarding OARs) in PCa SBRT^26^. Inherent SBRT characteristics, involving the delivery of high doses per fraction, underscore the critical need for meticulous couch shift observation to achieve superior dose distribution and minimize OAR exposure. Therefore, especially for patients with larger RVs or undergoing SBRT, increased vigilance during daily setup is crucial to optimize treatment outcomes^27^.

Achieving stringent control over patient positioning, thereby maintaining geometric uncertainties within exceptionally narrow error margins, offers the potential to transition from utilizing Van Herk’s more conservative PTV margin formula to the less encompassing Stroom’s formula^28,29^ which uses a smaller coefficient. PTV margin reduction, facilitated by minimized systematic and random errors, directly contributed to enhanced sparing of OARs while maintaining adequate target coverage, thereby optimizing the therapeutic ratio. Yartsev et al. recommend establishing quality assurance protocols, due to the lack of uniformity in margin selection among institutions was evident from their review. Our study directly contributes to the practical implementation by demonstrating the importance of checking the rectum’s distension and filling status prior to the treatment, which could be a critical factor influencing the position of the prostate^29^. Ultimately, our innovation aims to enhance the overall quality and safety of PCa radiotherapy.

Our study, while promising, has several limitations that warrant discussion and inform future research. The results were obtained from a single institution, highlighting the need for further validation with external datasets to confirm the model’s utility and generalizability in diverse clinical settings. To mitigate potential biases, future studies should consider pre-reported protocols, include a more diverse patient population, and employ blinded, randomized, and comparative designs. Exploring alternative model architectures or synthetic datasets could also lead to improved prediction accuracy.

We acknowledge the limitation that the rectal Region of Interest (ROI) shape may vary across different institutions, which could influence the generalizability of our volume data. Ambiguity in defining the rectosigmoid junction (RSJ) often introduces inter-institutional variability when measuring the rectal volume. To overcome this inconsistency and standardize volumetric assessment, we propose utilizing an anatomical landmark: the sigmoid take-off (STO). This point is best identified on a sagittal view where the sigmoid colon makes an abrupt ventral (anterior) turn, swinging clear of the sacrum/presacral fascia. This specific morphological feature precisely marks the physiological transition from the fixed support of the mesorectum to the mobile nature of the sigmoid mesocolon, offering a consistent, reliable standard for measurement^30^.

Future research shall include a critical, head-to-head comparison of predictive performance of different AI models and methods. This investigation could specifically benchmark classical statistical methods (e.g., Cox regression) against ML (e.g. random forest, support vector machine) and deep learning models. Current systematic reviews, such as that by Huang et al., suggest that the performance of classical statistics is often comparable to conventional ML approaches in medical prediction^31^. This suggests that a similar performance equivalence could extend to the ML versus DL comparison. Furthermore, a study by a research group is particularly noteworthy, demonstrating that deep adaptive learning provided better prediction performance than established algorithms like support vector machine (SVM), random forest (RF), and XGBoost. This finding strongly supports the viability of deep learning for accurate performance prediction^32^.

We investigated the table’s displacement by creating vectors and processing them through a deep learning pipeline. In the future, we recommend the observation of the displacement of the rectum mass gravity center, since it could carry additional information. Including the bladder in the volumetric analysis might offer additional benefits, as would examining rectal content characteristics, such as the distribution of gas and solid material. Furthermore, the promising rectum dose surface mapping technique recommended by Bouzaki et al. would be beneficial for toxicity prediction in future studies.^33^.

This study demonstrates a practical utility for deep learning in enhancing patient preparation for PCa radiotherapy. Our transformer-based model introduces a predictive capacity that allows the identification of high-risk setups based on simulation CT rectal volume (RV). By establishing an informed RV threshold, such as the 81.62 cm³ identified here, clinicians gain a new, quantifiable option to guide patient bowel and diet preparation before treatment. This proactive adjustment is expected to contribute to a more standardized and reproducible treatment environment, thereby supporting clinical workflow optimization by reducing the need for treatment-day repositioning or replanning.

## Conclusion

A novel transformer-based deep learning model was successfully developed and evaluated for predicting patient couch shifts in prostate cancer PCa radiotherapy. The central finding is the identification of a critical rectal volume threshold of 81.62 cm³ on planning CT scans. This threshold provides a data-driven criterion that enables clinical staff to initiate proactive patient preparation to modulate RV before the patient undergoes irradiation. This new predictive capability is a valuable tool for optimizing therapeutic delivery and contributes directly to the efficiency and rigor of the clinical workflow.

## Supplementary Information

Below is the link to the electronic supplementary material.

Supplementary Material 1

## Data Availability

The datasets generated during and/or analyzed during the current study are available from the corresponding author upon reasonable request.

## Abbreviations

AI: Artificial intelligence
cm³: Cubic centimeters
CBCT: Cone-beam computed tomography
CT: Computed tomography
DL: Deep learning
FFF: Filter-free
GCP: Guideline for Good Clinical Practice
IMRT: Intensity Modulated Radiation Therapy
ICH: International Council for Harmonisation of Technical Requirements for Pharmaceuticals for Human Use
Lat: Lateral
LINAC: Linear accelerator
Lng: Longitudinal
ML: Machine-learning
MV: Megavolts
OAR: Organ at risk
PTV: Planning target volume
PCa: Prostate cancer
RTT: Radiation therapy technologist
ROC AUC: Receiver Operating Characteristic Curve Area Under the Curve
RV: Rectal volume
SBRT: Stereotactic body radiation therapy
t-SNE: T-distributed Stochastic Neighbor Embedding
Vrt: Vertical
VMAT: Volumetric modulated arc therapy
