# Supplementary material for: Transformer-based prediction of radiotherapy couch shift risk in prostate cancer based on rectal volume
Source: Sci Rep. 2026 Mar 15;16:13565. doi: 10.1038/s41598-026-42276-7 (PMC13121726; doi:10.1038/s41598-026-42276-7)
Supplement: Supplementary file 1 — Supplementary Material 1 [file 41598_2026_42276_MOESM1_ESM.docx]

**Supplementary Materials:**


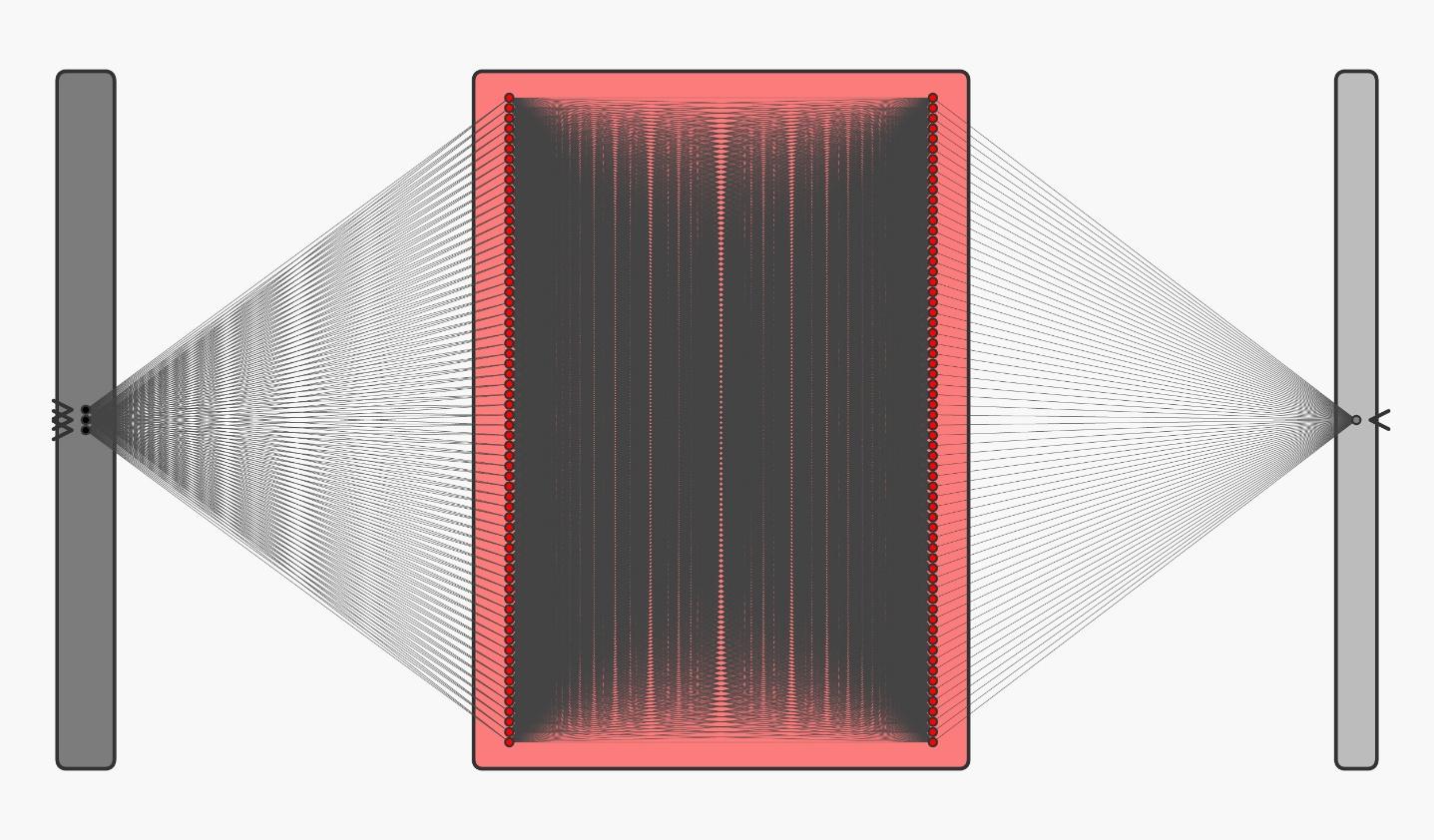


**Supplementary Figure 1**. Conceptual neural network schematic illustrating the transformation of patient longitudinal data. The Input Layer (dark green block, black nodes denoted by arrows) represents the three features (Delta_Rectum_Volume, Displacement_Magnitude, Rectum_Volume). These are processed through Hidden Layers (red block, red nodes), which conceptually represent the d_model = 64 dimensional embeddings and the operations of a Transformer Encoder, culminating in a single, comprehensive Output Layer (light grey block, grey node denoted by arrow) representing the embedded numerical value for each patient. Connections (grey lines) depict data flow, and transparent colored blocks visually delineate the layers.


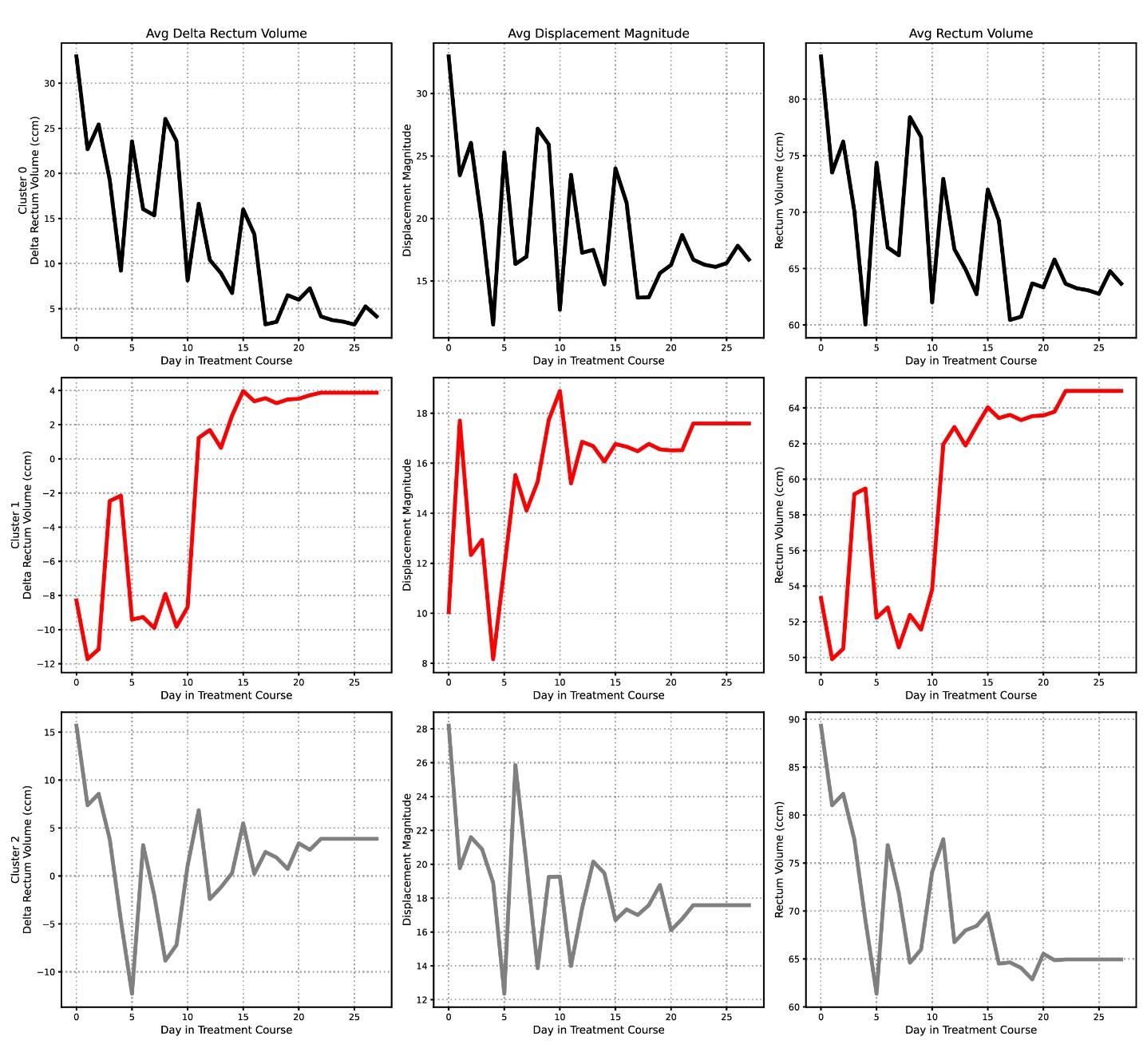


**Supplementary Figure 2.** Average feature trajectories for each identified cluster across treatment days. Each row represents a distinct patient cluster (Cluster 0, Cluster 1, Cluster 2), and each column displays the average trajectory for a specific feature: Delta_Rectum_Volume, Displacement_Magnitude, and Rectum_Volume. Treatment day is indicated by the x-axis, while the y-axis shows the unscaled feature values.


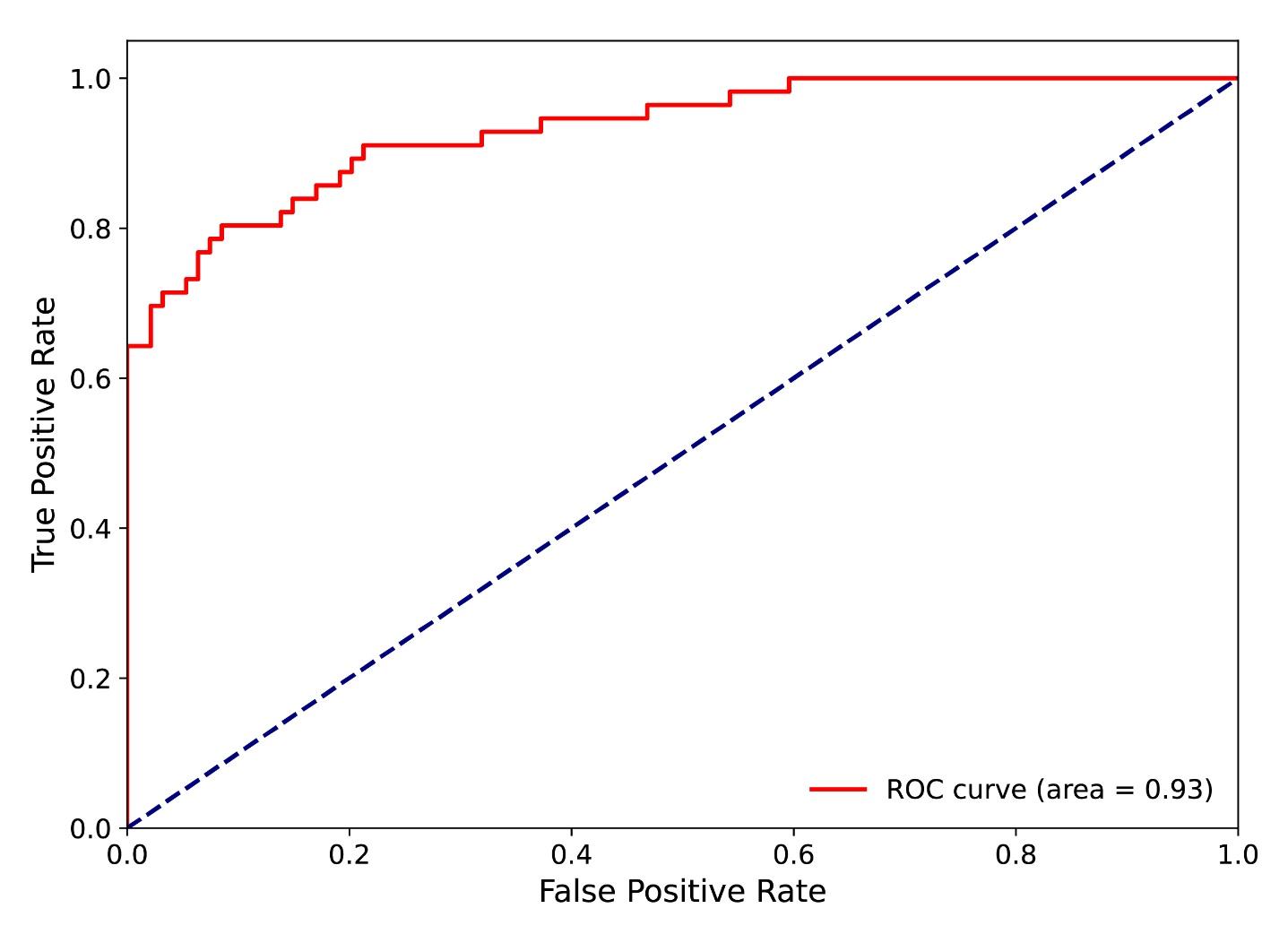


**Supplementary Figure 3**. ROC curve measures the performance of the initial rectal volume (RV) in Cluster 2 vs Clusters 0-1.
